# ADHD coaching since the COVID-19 pandemic: Is there a new wave of ADHD coaches?

**DOI:** 10.3389/fpsyt.2026.1841020

**Published:** 2026-09-03

**Authors:** Elias D. Graham, Melissa R. Dvorsky, Carlos E. Yeguez, Jillian K. Holbrook, Margaret H. Sibley

**Affiliations:** 1 Department of Psychology, University of Washington, Seattle, WA, United States; 2 Department of Psychiatry & Behavioral Sciences, George Washington University School of Medicine and Health Sciences, Washington, DC, United States; 3 Center for Health Outcomes Research and Delivery Science, Children’s National Research Institute, Washington, DC, United States; 4 Department of Psychiatry & Behavioral Sciences, University of Washington School of Medicine, Seattle, WA, United States; 5 Center for Child Health, Behavior, and Development, Seattle Children’s Research Institute, Seattle, WA, United States

**Keywords:** ADHD, ADHD coaching, COVID-19 pandemic, psychosocial intervention, public health trends, secondary data analysis

## Abstract

The COVID-19 pandemic saw an increased demand for ADHD psychosocial treatment, which exceeded capacity of traditional healthcare providers. Online ADHD coaching may have become a source of support for individuals seeking support during this period. This study investigates trends in ADHD coaching between coaches who began pre- versus post-COVID-19 pandemic. We conducted secondary analyses (*N* = 465) of the U.S. National Survey on ADHD Coaching, which used a purposive electronic survey and snowball sampling to select self-identified ADHD coaches with at least one client in the U.S. in the past year. We compared pre-pandemic (*n* = 182) and post-pandemic (*n* = 283) coaches across survey items. Post-pandemic coaches were younger (*t* = 9.48, *p* < .001), more likely to self-identify as having ADHD (χ^2^ = 9.01; *p* = .003), and more professionally isolated, with lower rates of referring clients to CBT providers (χ^2^ = 8.63; *p* = .003) or medication prescribers (χ^2^ = 27.60, *p* < .001), and lower rates of healthcare licensure (χ^2^ = 5.90; *p* = .015) or professional liability insurance (χ^2^ = 11.1; *p* < .001). Post-pandemic coaches were less likely to discuss clinical topics, including medication adherence (χ^2^ = 18.06; *p* < .001), substance use or addictions (χ^2^ = 15.86; *p* < .001), and suicide, abuse, or harm to self/others (χ^2^ = 16.11; *p* < .001). Following the COVID-19 pandemic, pre-pandemic coaches were more likely to see clients virtually (53.85%), have a larger caseload (28.57%), and practice outside of their local region (30.22%). Differences between pre- and post-pandemic ADHD coaches may indicate where the field is headed and should be considered when researching ADHD coaching, designing training standards, and evaluating need for regulatory structures, clinical oversights, and clarified professional boundaries between ADHD coaching and psychotherapy.

## 1 Introduction

The COVID-19 pandemic witnessed a spike in attention-deficit/hyperactivity disorder (ADHD) medication demand, nonmedical support seeking, and online activity continuing today (1). In recent years, help-seeking for ADHD has increased (2, 3), possibly due to changes in the DSM-5’s diagnostic criteria and increased online information sharing about ADHD (4). Though 7.8% of U.S. adults have been diagnosed with ADHD, approximately 40% are not receiving clinical treatments (1). Evidence-based treatments for ADHD include medications (stimulant/non-stimulant) and cognitive behavioral therapy (CBT), traditionally delivered by licensed psychologists or mental health providers (5–7).

Alongside well-publicized increased demands for medication has come increased demand for psychosocial support (1). Lockdown restrictions at the pandemic’s outset drove a pivot to online mental health support, including via social media (8, 9). While healthcare providers were quick to adapt to telehealth practices, demands for online mental health care exceeded capacity of licensed psychosocial care practitioners (10, 11). Options for individuals with ADHD were especially limited, as most psychotherapists do not offer psychotherapy to adults whose primary presenting condition is ADHD [compared to similarly prevalent conditions; (12)]. This disparity is unsurprising as some psychosocial providers report frustration with treatment-interfering behaviors commonly associated with ADHD (13, 14).

Limited availability of ADHD specialty psychosocial care may have contributed to increasing interest in and visibility of online ADHD coaching (15, 16). ADHD coaching emerged from the life coaching movement in the 90s but is unique in its focus on ADHD. Public-facing information on ADHD coaching describes the service as client-centered, goal-oriented, and collaborative, providing support and education on ADHD (17). Even when CBT for ADHD is accessible, clients may intentionally seek out ADHD coaching due to its unique model which often includes check-ins throughout the week and peer support from a coach who also has ADHD (18).

Despite its rising popularity, there are nearly no systematic evaluations of the effectiveness and safety of ADHD coaching, particularly in its modern forms (19–23). Unlike CBT for ADHD delivered by psychotherapists, ADHD coaches are not required to receive formal training or licensure, and there are no educational requirements, supervision expectations, geographic restrictions, or legal guidelines regulating their practices (24). While most ADHD coaches appear to be non-clinical practitioners who also have ADHD themselves, some are concurrent healthcare providers (18). Many adults with ADHD have tried ADHD coaching, reporting higher satisfaction with it than medication or CBT (25). Given the limited availability of specialty psychosocial ADHD care, ADHD coaching should be further studied to determine how training, credentialing, clinical consultation pathways, ethical standards, and referral practices relate to effectiveness and safety.

Recently, we conducted the U.S. National Survey on ADHD Coaching to increase understanding of the current landscape of ADHD coaching in the United States. We reported an influx of ADHD coaches joining the workforce in the U.S. (18), with a notable spike in workforce entry at the beginning of the COVID-19 pandemic, mirroring similar trends for ADHD medications (2). In the reported sample [*N* = 465; (18)], the average number of ADHD coaches joining the workforce each year was about five times higher between 2019 and 2024 (*M* = 53.5) than between 2010 and 2018 (*M* = 10.67). The pivot to online (versus in-person) ADHD coaching likely lowered barriers to and increased the appeal of ADHD coaching workforce entry. Because there are no legal requirements to become an ADHD coach, relatability and charisma can be commodified for marketing purposes, overshadowing the importance of educational level and employment experience in healthcare sectors (9, 18). The unique digital and pandemic-related factors contributing to ADHD coaching’s recent rise raise questions about whether ADHD coaches entering the workforce during or after the COVID-19 pandemic differ systematically from those who began pre-pandemic.

Several universals emerged from our study regardless of workforce entry date (18). ADHD coaches were largely self-employed business owners operating outside the U.S. healthcare system, who worked virtually. Most had no background in mental health and instead completed an ADHD coach-led curriculum. However, there was also substantial heterogeneity between coaches that may be explained by = ADHD coaching workforce entry date. Herein, we investigate how post-pandemic and pre-pandemic ADHD coaches may differ to better understand shifts in the field in the wake of the COVID-19 pandemic.

We offer several hypotheses about mechanisms of pre- versus post-pandemic ADHD coach differences. First, we hypothesize generational differences given the migration of the ADHD coaching profession to online spaces. In these spaces, ADHD-focused lived experience content is pervasive and popular (9), which may facilitate dissemination of ADHD coaching advertisements to younger user demographics and to those who self-identify with ADHD (26, 27). Given low barriers to ADHD coaching workforce entry, increased visibility over digital media could incur a wave of ADHD coaches who are less likely to hold an advanced degree, healthcare licensure, or have prior experience in healthcare fields, but may be more demographically diverse themselves and serve more diverse client bases (28).

Post-pandemic ADHD coaches also may display lower professional engagement due to rapid workforce entry and instant visibility with potential clients via informal connections within the ADHD social media community, rather than traditional methods such as formally joining an ADHD coaching organization, attending ADHD coach training programs, and fostering professional relationships with the healthcare and educational sectors to receive direct referrals. Given the newfound ease of launching an ADHD coaching practice, post-pandemic ADHD coaches may also be more likely to launch part-time, smaller-scale operations, mirroring trends in increasing entrepreneurships over the pandemic (29, 30).

For similar reasons, we expect post-pandemic ADHD coaches to rely more on lived experiences in their practices and adopt the neurodiversity movement that is dominant on social media (31). Their lower connectedness to the ADHD coaching community may come at a cost of reduced socialization to the risks of their field, including a need to maintain a clear boundary between coaching and psychotherapy by avoiding clinical topics such as medication adherence, substance use, or self-harm, as well as the importance of obtaining professional liability insurance (18). They also may be less likely to use pre-existing ADHD coaching resources, such as structured intervention materials.

Practice shifts also may have occurred within individuals among pre-pandemic coaches. For example, after the onset of the pandemic, pre-pandemic coaches may have pivoted practices to mirror overall ADHD healthcare trends (1): seeing more clients virtually, seeing more clients outside of their local region (due to increased virtual options), and using more online/social media advertising methods. With these facilitators of practice expansion in place, they also may have expanded their caseload due to increased reach and higher demand for ADHD services (2).

The current study offers secondary data analyses of the U.S. National Survey on ADHD Coaching with attention to between and within group differences in ADHD coaches prior to and after the onset of the COVID-19 pandemic. We tested our 1) generational, 2) professional engagement, and 3) session content hypotheses (see **Supplementary Table 1** in the **Supplementary Materials**) across 37 indicators of ADHD coach characteristics and their practices.

## 2 Methods

### 2.1 Procedure

The UW Human Subjects Division IRB approved this study (ID: STUDY00020664). All procedures were conducted in accordance with ethical guidelines for human subjects research. Participants provided digital consent using an anonymous link to access study materials on Qualtrics. No compensation was offered for survey completion.

Participants were recruited over six months from October of 2024 to April of 2025. Given the ADHD coaching workforce’s unknown size and the survey’s exploratory focus on surveillance, we utilized purposive and snowball sampling to recruit any individuals self-identifying as an ADHD coach. We utilized a multipronged outreach strategy to achieve as large and representative a sample of ADHD coaches as possible (e.g., emailing professional ADHD coaching organization listservs, social media postings by organizations likely to include ADHD coaches, recruiting in person at ADHD conferences, sending direct emails or DMs to ADHD coaches identified through online searches, etc.). For more information about procedures, participant recruitment, and survey methodology, see Sibley et al. (18).

### 2.2 Data integrity

To protect our survey from fraudulent responses, we implemented several data integrity measures consistent with best practices for ensuring data validity as well as bot prevention and detection (32–36). These procedures are described in detail in the **Supplementary Materials**.

### 2.3 Participants

To be eligible, participants must have delivered ADHD coaching services to at least one client located in the United States, U.S. territories, or U.S. military bases within the past 12 months. Outreach and recruitment strategies resulted in 792 self-identified ADHD coaches receiving the survey link, 670 of whom accessed the survey. Of the 670 participants who accessed the survey, 156 (23.3%) were excluded for not meeting study criteria. For information about participant flow and reasons for study exclusion, including full consort flow diagram, please see Sibley et al. (18). We included 465 participants in the present analysis who reported coaching start date (see **Supplementary Materials**).

### 2.4 Measure

The U.S. National Survey on ADHD Coaching (18) was an online survey, consisting of 50 quantitative and 10 qualitative questions divided into 5 main subcategories: coaching practice, pathway to ADHD coaching, demographics and coaching background, coaching style, and inter-professional connections, and an optional section containing the qualitative questions about coaches’ views and perspectives. For full survey and information about survey development, see Sibley et al. (18).

### 2.5 Analytic plan

Responses were compared for ADHD coaches who joined the coaching workforce before versus after March 2020 (pre-COVID-19 pandemic: *n* = 182; post-COVID-19 pandemic: *n* = 283) across 37 study variables drawn from the quantitative portion of the survey. Excluded survey items were omitted due to insufficient heterogeneity (e.g., 97.15% of coaches practice virtually).

Data from all 465 participants who reported the date they began coaching were analyzed, including respondents who only partially completed the survey (*n* = 5). Participants were included in our subsample if they reached the question asking about their coaching start date and answered at least one outcome measure question. For continuous variables, means and standard deviations were used to describe the variables, and pre- and post-pandemic coaches were compared using independent-samples *t*-tests with Cohen’s *d* as an estimate of effect size. Because parametric analyses assume that the variables approximate a normal distribution, the normality of all continuously scaled variables was additionally assessed using the Shapiro-Wilk test (37), with *p*-values above.05 indicating normality. For non-normally distributed continuous variables, medians and ranges were additionally used to describe the variables, and Mann-Whitney *U*-tests were additionally reported with rank-biserial correlations (*r_rb_*) as non-parametric estimates of effect size (see **Supplementary Materials**). For categorical variables, responses were described using relative values (%) and absolute values (*n*) and compared using independent chi-square tests with odds ratio (*OR*) as an estimate of effect size.

Due to the large number of tests run, separate analyses were performed within each domain reported in the original study [e.g., demographics, service delivery, coaching practices; (18)], and a Benjamini-Hochberg correction was applied to correct for experiment-wise error rate (38). *Post hoc* pairwise comparisons were performed for statistically significant chi-square tests involving more than two categories with a Benjamini-Hochberg correction applied. To discourage overreliance on null-hypotheses, statistical significance testing confidence intervals were provided around all effect sizes of interest to make use of the null regions framework (39). All analyses were performed using jamovi statistical software (40).

## 3 Results

### 3.1 ADHD coach demographics

Demographic characteristics of ADHD coaches and statistical test results are summarized in **Table 1**. Compared to ADHD coaches who joined the coaching workforce pre-pandemic, those who joined the workforce post-pandemic were more likely to self-identify as having ADHD (post: 77.58%, pre: 64.84%; χ^2^ = 9.01; *p* = .003; *OR* = 1.88), more likely to have received a formal ADHD diagnosis (post: 63.35%, pre: 52.20%; χ^2^ = 5.67; *p* = .017; *OR* = 1.58), and more likely to have received ADHD coaching as a client (post: 49.47%, pre: 36.81%; χ^2^ = 7.16; *p* = .008; *OR* = 1.68). Post-pandemic ADHD coaches also tended to be younger (post: *M* = 47.61, pre: *M* = 57.18; *p* < .001, *d* = -0.91) and were more likely to identify as a racial/ethnic minority (post: 15.90%, pre: 8.24%; χ^2^ = 5.78; *p* = .016; *OR* = 2.11). Finally, post-pandemic coaches were less likely to have an advanced degree (post: 61.84%, pre: 73.63%; χ^2^ = 6.91; *p* = .009; *OR* = 0.58), to have healthcare licensure (post: 11.66%, pre: 19.89%; χ^2^ = 5.90; *p* = .015; *OR* = 0.53), or to have worked in a mental health related field directly prior to becoming an ADHD coach (post: 7.45%, pre: 15.93%; χ^2^ = 8.29; *p* = .004; *OR* = 0.42). No other demographics differed to a statistically significant degree across coaching groups (**Table 1**).

**Table 1:** Descriptives and frequencies of ADHD coach demographics characteristics.

Variable	Coaches entering the workforce % (*n*)		Omnibus effect	*p*	Effect size (95% CI)
	Pre-pandemic	Post-pandemic			
Lived experiences					
Self-identify as having ADHD	64.84% (118)	77.58% (218)	*X^2^ =* 9.01	0.003*	1.88 (1.24, 2.84)
Received formal ADHD diagnosis^a^	52.20% (95)	63.35% (178)	*X^2^ =* 5.67	0.017*†	1.58 (1.08, 2.31)
Received ADHD coaching as client^a^	36.81% (67)	49.47% (139)	*X^2^ =* 7.16	0.008*†	1.68 (1.15, 2.46)
Family member with ADHD	76.92% (140)	77.94% (219)	*X^2^ =* 0.07	0.799	1.06 (0.68, 1.65)
Close friends or romantic partners with ADHD	52.75% (96)	65.13% (183)	*X^2^ =* 7.07	0.008*†	1.67 (1.14, 2.45)
Teaching or working professionally with individuals with ADHD	64.29% (117)	68.68% (193)	*X^2^ =* 0.97	0.326	1.22 (0.82, 1.81)
Gender					
Woman (vs all other)	80.77% (147)	79.86% (226)	*X^2^ =* 0.06	0.810	0.94 (0.59, 1.51)
Man (vs all other)	15.93% (29)	14.49% (41)	*X^2^ =* 0.18	0.670	0.89 (0.53, 1.50)
Age (M [SD])^b^	57.18 (10.91)	47.61 (47.5)	*t* = -9.48	<.001*	*d* = -0.91
White, non-Hispanic	91.76% (167)	84.10% (238)	*X^2^ =* 5.78	0.016*†	0.48 (0.26, 0.88)
Education Level/Degree^b^			*X^2^ =* 6.91	0.009*	0.58 (0.39, 0.87)
Bachelor’s degree or less	26.37% (48)	38.16% (108)			
Advanced degree	73.63% (134)	61.84% (175)			
Healthcare licensure	19.89% (36)	11.66% (33)	*X^2^ =* 5.90	0.015*	0.53 (0.32, 0.89)
Job sector prior to coaching ^b^					
Education	29.67% (54)	32.62% (92)	*X^2^ =* 0.45	0.504	1.15 (0.77, 1.72)
Business	10.44% (19)	11.35% (32)	*X^2^ =* 0.09	0.760	1.10 (0.60, 2.00)
Mental health	15.93% (29)	7.45% (21)	*X^2^ =* 8.29	0.004*	0.42 (0.23, 0.77)
Professional engagement ^b^					
Completed an ACO-endorsed ADHD Coach-Led Training Curriculum^c^	37.36% (68)	37.46% (106)	*X^2^ =* 0.01	0.984	1.00 (0.68, 1.48)
Member of a Professional ADHD Coaching Organization^d^	59.89% (109)	56.89% (161)	*X^2^ =* 0.41	0.522	0.88 (0.61, 1.29)

Continuous variables were compared using independent-samples *t*-tests and reported as *mean* (*standard deviation*) with Cohen’s *d* reported for effect size. Categorical variables were compared using independent chi-square tests and reported as relative and absolute values, % (*n*), with odds ratios reported as an estimate of effect size. ^a^ Items were only asked to those who self-identify as having ADHD. ^b^ Category is mutually exclusive, whereas all other responses under categories in the table above are not mutually exclusive, and respondents could select all that apply. ^c^ Individuals who endorsed completed one of the ADHD training courses listed on the ACO website. ^d^ Includes membership in ACO or PAAC. ACO=ADHD Coaching Organization. PAAC=Professional Association or ADHD Coaches. † Finding was no longer significant following sensitivity analysis adding age as a covariate.

### 3.2 ADHD coach service delivery

Compared to pre-pandemic coaches, post-pandemic coaches were less likely to be covered by professional liability insurance (post: 55.48%, pre: 70.88%; χ^2^ = 11.1; *p* < .001; *OR* = 0.51; see **Table 2**). When looking at where ADHD coaches sourced their clients, post-pandemic coaches were less likely to receive referrals from healthcare providers (post: 57.95%, pre: 76.37%; χ^2^ = 16.56; *p* < .001; *OR* = 0.43), less likely to receive referrals from educational sectors (post: 21.91%, pre: 37.92%; χ^2^ = 14.02; *p* < .001; *OR* = 0.46), and less likely to advertise their services through paid advertisements (post: 14.84%, pre: 28.57%; χ^2^ = 12.95; *p* < .001; *OR* = 0.44). Regarding populations served, post-pandemic coaches were less likely to provide coaching to college students (post: 63.96%, pre: 74.59%; χ^2^ = 5.74; *p* = .017; *OR* = 0.60) and were more likely to serve mostly women/girls (post: 37.10%, pre: 19.89%; χ^2^ = 15.46; *p* < .001). Finally, post-pandemic coaches actively saw fewer clients (post: *Mdn* = 8, pre: *Mdn* = 15; *p* < .001, *d* = 0.36) and saw clients for fewer months (post: *Mdn* = 6, pre: Mdn = 9; *p* < .001, *d* = 0.45), spent fewer direct hours coaching (post: *Mdn* = 7, pre: *Mdn* = 12; *p* < .001, *d* = 0.41), and charged less for individual sessions (post: *Mdn* = 125, pre: *Mdn* = 150; *p* = .001, *d* = 0.32). No other delivery characteristics differed to a statistically significant degree across coaching groups (**Table 2**).

**Table 2:** Descriptives and frequencies of ADHD coach service delivery characteristics.

Variable	Coaches entering the workforce % (*n*)		*X^2^*	*p*	*OR* (95% CI)
	Pre-pandemic	Post-pandemic			
*Delivers coaching across state lines*	41.32% (157)	58.68% (223)	3.77	0.052	0.59 (0.34, 1.01)
*Delivers coaching across international borders*	44.75% (81)	38.16% (108)	1.99	0.159	0.76 (0.52, 1.11)
*Delivers coaching in-person*	46.41% (84)	43.11% (122)	0.49	0.485	0.87 (0.60, 1.27)
*Accepts outside funding (e.g., FSA, HAS, Client’s employer, health insurance)*	27.47% (50)	22.62% (64)	1.41	0.235	0.77 (0.50, 1.18)
*Professional Liability Insurance Coverage* ^a^	70.88% (129)	55.48% (157)	11.1	<.001*	0.51 (0.34, 0.76)
Source of clients					
Advertisement in online directory	76.92% (140)	67.49% (191)	4.80	0.028	0.62 (0.41, 0.95)
Referrals from healthcare providers	76.37% (139)	57.95% (164)	16.56	<.001*	0.43 (0.28, 0.65)
Social media marketing	46.70% (85)	50.18% (142)	0.53	0.465	1.15 (0.79, 1. 67)
Referrals from educational sector	37.91% (69)	21.91% (62)	14.02	<.001*	0.46 (0.30, 0.69)
Paid advertisements	28.57% (52)	14.84% (42)	12.95	<.001*	0.44 (0.28, 0.69)
Word of mouth	18.13% (33)	19.08% (54)	0.07	0.798	1.33 (0.83, 2.17)
Populations served					
College students	74.59% (135)	63.96% (181)	5.74	0.017*	0.60 (0.40, 0.91)
Adolescents (age 12-17)	47.51% (86)	43.82% (124)	0.61	0.435	0.86 (0.59, 1.25)
Children under 12	19.34% (35)	21.56% (61)	0.33	0.565	1.15 (0.72, 1.83)
I mostly serve White individuals	70.72% (128)	68.91% (195)	0.17	0.679	0.92 (0.61, 1.38)
I mostly serve women/girls	19.89% (36)	37.10% (105)	15.46	<.001*	2.38 (1.53, 3.68)
I mostly serve adults with at least a Bachelors	62.43% (113)	62.90% (178)	0.01	0.919	1.02 (0.69, 1.50)
	Coaches entering the workforce *Med* (min-max)		*t*	*p*	*d*
	Pre-pandemic	Post-pandemic			
Number of clients on caseload	15 (1-200)	8 (0-300)	3.77	<.001*	0.36
Length of sessions (minutes)	55 (20-90)	55 (20-120)	-1.82	0.069	-0.17
Number of sessions per month per client	4 (2-160)	4 (1-100)	0.70	0.483	0.07
Duration of active services per client (in weeks)	9 (1-75)	6 (1-48)	4.46	<.001*	0.45
Total weekly direct client hours	12 (0-50)	7 (0-60)	4.28	<.001*	0.41
Total weekly indirect hours	5.5 (0-45)	5 (0-102)	0.07	0.947	0.01
Individual session rate/fees^b^	150 (0-750)	125 (0-500)	3.30	0.001*	0.32

^a^ This category is mutually exclusive, whereas all other responses under categories in the table above are not mutually exclusive, and respondents could select all that apply. ^b^ A total of *n* = 178 pre-pandemic coaches and *n* = 279 post-pandemic coaches reported a dollar amount for their individual session rate/fees and in each group one case reported fees of $3,000 and $2,500 or more, respectively, which were Winsorized to the next highest value ($750 and $500 respectively) to reduce the impact of these outliers in positively skewing the data.

### 3.3 ADHD coaching practices

ADHD coaching practice characteristics and statistical test results are summarized in **Table 3**. Compared to pre-pandemic coaches, post-pandemic coaches were less likely to utilize structured intervention materials (post: 53.57%, pre: 67.03%; χ^2^ = 8.26; *p* = .004; *OR* = 0.57). Regarding clinical topics covered, post-pandemic coaches were less likely to discuss ADHD medication adherence (post: 71.07%, pre: 87.91%; χ^2^ = 18.06; *p* < .001; *OR* = 0.34), substance use or addictions (post: 45.36%, pre: 64.29%; χ^2^ = 15.86; *p* < .001; *OR* = 0.46), or to have clients disclose sensitive information such as suicide, abuse, and/or harm to self or others (post: 33.93%, pre: 52.75%; χ^2^ = 16.11; *p* < .001; *OR* = 0.46). Post-pandemic coaches were also less likely to interpret outside evaluations (post: 27.14%, pre: 22.51%; χ^2^ = 14.82; *p* < .001; *OR* = 0.46). Finally, post-pandemic coaches were less likely to refer clients to medication prescribers (post: 51.43%, pre: 75.82%; χ^2^ = 27.60; *p* < .001; *OR* = 0.34) or CBT providers (post: 45.36%, pre: 59.34%; χ^2^ = 8.63; *p* = .003; *OR* = 0.57). No other coaching practice characteristics differed to a statistically significant degree across coaching groups (**Table 3**).

**Table 3:** Descriptives and frequencies of ADHD coaching practices.

Variable	Coaches entering the workforce % (*n*)		*X^2^*	*p*	*OR* (95% CI)
	Pre-pandemic	Post-pandemic			
Receipt of supervision or informal consultation					
Peer consultation from another coach	23.08% (42)	23.93% (67)	0.04	0.833	1.05 (0.67, 1.63)
Informal consultation from a licensed provider	59.89% (109)	69.29% (194)	4.31	0.038	1.51 (1.02, 2.23)
Clinical supervision from a licensed provider	9.34% (17)	9.29% (26)	< 0.01	0.984	0.99 (0.52, 1.89)
Conceptualize ADHD as…					
Neurodevelopmental disorder	45.60% (83)	49.11% (138)	0.54	0.461	1.15 (0.79, 1.67)
Mental health condition	18.13% (33)	21.71% (61)	0.87	0.350	1.25 (0.78, 2.01)
Psychiatric disorder	7.69% (14)	7.47% (21)	0.01	0.931	0.97 (0.48, 1.96)
Mental disorder	4.40% (8)	7.12% (20)	1.44	0.230	1.67 (0.72, 3.87)
Structured Intervention Materials Utilized			8.26	0.004*	0.57 (0.38, 0.83)
Curriculum or protocol developed by self or someone else	67.03% (122)	53.57% (150)			
None	32.97% (60)	46.43% (130)			
Clinical concerns: topics covered ^a^					
ADHD medication adherence	87.91% (160)	71.07% (199)	18.06	<.001*	0.34, (0.20, 0.56)
Substance use or addictions	64.29% (117)	45.36% (127)	15.86	<.001*	0.46 (0.31, 0.68)
Trauma	53.30% (97)	45.71% (128)	2.54	0.111	0.74 (0.51, 1.08)
Suicide, abuse, and/or harm to self or others	52.75% (96)	33.93% (95)	16.11	<.001*	0.46 (0.31, 0.67)
Use of measurement-based care					
Progress monitoring	44.51% (81)	45.36% (127)	0.03	0.857	1.04 (0.71, 1.51)
Interpret outside evaluations	44.51% (81)	27.14% (76)	14.82	<.001*	0.46 (0.31, 0.69)
No assessment	28.57% (52)	32.50% (91)	0.80	0.372	1.20 (0.80, 1.81)
Collect client satisfaction ratings	26.37% (48)	31.43% (88)	1.36	0.244	1.28 (0.84, 1.94)
Conducts ADHD diagnostic assessments	15.93% (29)	9.29% (26)	4.65	0.031	0.54 (0.31, 0.95)
Outgoing referrals to healthcare					
A (mental) healthcare provider to obtain formal diagnosis	84.07% (153)	75.71% (212)	4.64	0.031	0.59 (0.36, 0.96)
Medication prescribers	75.82% (138)	51.43% (144)	27.60	<.001*	0.34 (0.22, 0.51)
Cognitive behavioral therapy providers	59.34% (108)	45.36% (127)	8.63	0.003*	0.57 (0.39, 0.83)
Holistic, naturopathic, or other healing arts professionals	26.92% (49)	17.86% (50)	5.38	0.020	0.59 (0.38, 0.92)

All ADHD coaches were asked all coaching practice questions, regardless of the age group they reported serving in their caseload. ^a^ Response choices ranged from “none” (0), “some” (1), and “a lot” (2), and “some” and “a lot” were collapsed together to describe any use for the frequency/percentage.

### 3.4 COVID-19 impact

Most pre-pandemic ADHD coaches reported that their practice permanently changed due to the COVID-19 pandemic (62.64%; *n* = 114; **Table 1**). More specifically, 30.22% (*n* = 55) of pre-pandemic coaches responded that they now see clients outside of their local region, 53.85% (*n* = 98) now see clients virtually, 14.29% (*n* = 26) now use more online/social media advertising methods, 28.57% (*n* = 52) have a larger caseload, 11.54% (*n* = 21) now work for themselves, and 6.59% (*n* = 12) now create content or materials for other coaches (**Table 4**).

**Table 4:** COVID-19 permanently altering pre-pandemic ADHD coaching practices.

In what ways did the COVID-19 pandemic permanently alter your coaching practice?	% (n)
I now see clients outside of my local region	30.22% (55)
I now see clients virtually	53.85% (98)
I now use more online/social media advertising methods	14.29% (26)
I now have a larger caseload or longer waitlist	28.57% (52)
I now work for myself	11.54% (21)
I now create content or materials for other coaches	6.59% (12)
My practice did not change permanently due to the COVID-19 pandemic	37.36% (68)

These questions were asked as follow-up questions to coaches who joined the ADHD coaching workforce prior to the COVID-19 pandemic (*n* = 182).

### 3.5 Sensitivity analysis

For sensitivity analyses considering the effect of age, see **Supplementary Materials**.

## 4 Discussion

We sought to characterize a previously reported spike in ADHD coach workforce entry at the onset of the COVID-19 pandemic (9) and how the pandemic shifted pre-pandemic ADHD coaching practices. Very different profiles of pre-pandemic versus post-pandemic ADHD coaches emerged. As hypothesized, post-pandemic coaches were younger, more likely to have ADHD lived experience, and were more professionally isolated, with less connection to the healthcare and educational sectors. However, post-pandemic coaches did not report less connection to ADHD coaching organization membership or training, and there was mixed support for our hypotheses regarding professional risks, with lower rates of liability insurance among post-pandemic coaches, but less overstepping into the scope of clinical care compared to pre-pandemic coaches.

We found partial support for our generational differences hypothesis, as post-pandemic coaches tended to be younger, more likely to self-identify as having ADHD, and less likely to hold an advanced degree or healthcare licensure, though no statistically significant differences were found regarding social media marketing. Despite a few statistically significant differences in demographic characteristics, both coaching groups overall reported high self-identification with ADHD, and tended to be White, to identify as a woman, and did not endorse previous experience in a mental health field or current healthcare licensure.

The higher levels of personal connection to ADHD reported by post-pandemic coaches were paired with greater emphasis on personal lived experience during sessions—hallmarks of a peer support model. This finding suggests that modern ADHD coaching may be shifting towards peer-based support [e.g., empathy, shared experiences, client empowerment, building hope, and reducing self-stigmatization; (41)]. This finding may have facilitated ADHD coaching’s rapid post-pandemic popularity among consumers by enhancing perceived credibility and ability to successfully relate with clients. However, it is unclear whether the newer wave of coaches is at an increased risk of overgeneralizing their experiences with ADHD to those of their clients, as has been previously questioned [e.g., inappropriate disclosures, projection, countertransference; (42)]. This higher rate of ADHD self-identification among post-pandemic ADHD coaches additionally aligns with increasing rates of disability disclosure among the more general life coaching population, as well as increasing rates of coaches who are younger and hold diverse racial/ethnic identities (28). There also appear to be post-pandemic shifts in practice size and scope (i.e., more part-time work), smaller caseloads, and more limited forms of ADHD coaching. This may be explained by either the increased feasibility of launching smaller-scale ADHD coaching businesses or difficulty achieving a full-time caseload as a new ADHD coach.

We also found partial support for our professional engagement and session content hypotheses, as post-pandemic coaches were less professionally engaged with external healthcare providers, but not other ADHD coaches, and contrary to initial hypotheses, post-pandemic ADHD coaches were less likely to cover clinical topics in sessions. The professional isolation of post-pandemic ADHD coaches may both protect against drift outside one’s scope of practice (e.g., interpreting clinical evaluations and discussing clinical topics) and prevent important inter-professional connections that promote appropriate and holistic support for ADHD, such as referring to healthcare providers when need arises. It is encouraging that newer coaches may be maintaining clearer boundaries with psychotherapy by avoiding clinical topics such as self-harm, substance use, and medication adherence (43–45). However, there is also an indication that more recent coaches have limited awareness of important professional practices, such as considering their appropriateness to support geographically distant clients or obtaining professional liability insurance coverage designed to reduce their own liability or negative consequences of adverse events that may occur (46). Their narrower form of ADHD coaching (i.e., less likely to discuss comorbidities) may indicate that post-pandemic ADHD coaches view themselves as fulfilling a narrower role or may avoid more complex cases that inevitably lead to discussion of comorbidities. The pre-pandemic generation of coaches, who have been in practice longer, may be more confident in their abilities to provide support for co-occurring concerns, demonstrating greater overlap with clinical interventions such as CBT.

Despite the fact that pre-pandemic coaches indicated lasting practice changes in the wake of the pandemic (i.e., pivoting to virtual sessions, increased geographic reach), their modern practices still demonstrate differences, indicating that there is something distinctly different about the way newer versus historical coaches are currently practicing. Beyond trends among newer ADHD coaches, we note that scalability and client access appear to be ubiquitously expanding in the ADHD coaching workforce, raising important questions about statutory regulations. We encourage public health conversations between ADHD coaches and mental health stakeholders to build cross-professional relationships between ADHD coaches and therapists, and increase training opportunities to aid ADHD coaches in managing professional risks (i.e., emphasizing holding liability insurance and restricting practice to within US jurisdiction for post-pandemic coaches, and providing resources for interfacing with healthcare systems when sensitive clinical topics arise, particularly for pre-pandemic ADHD coaches).

### 4.1 Limitations

Although this study followed many best practices by employing a comprehensive and inclusive multimodal recruitment strategy, developing its survey in close collaboration with members of the ADHD coaching community, and implementing rigorous data integrity safeguards to ensure quality and reliability of responses, it possesses limitations. Sensitivity analyses revealed that in some cases, the effect of workforce entry date could not be disentangled from a coach’s age. Particularly for the variables of receiving an ADHD diagnosis, receiving coaching as a client, and participant race, the sensitivity analysis revealed that, in addition to timeframe, coach age may be contributing to some differences between coaching groups.

Additionally, as the true coaching population is unknown, it is possible the present sample is not fully representative of all ADHD coaches in the U.S., which could impact generalizability of results. There is additional risk of selection bias since survey respondents may be more likely to engage in online activity (e.g., follow ADHD community social media accounts, engage in ADHD community hubs) or be members of professional organizations we recruited through. They may also be more likely to demonstrate trust in researchers. Given limited demographic diversity of coaches in this sample (most of whom were middle-aged White women), results may not generalize to coaches outside this demographic. It is also unclear whether these results vary as a function of coach training background or credentialing (by an ADHD or life coaching organization).

### 4.2 Future directions

Future research that examines the efficacy, safety, and long-term outcomes of ADHD coaching might include pre- versus post-pandemic workforce entry as an important intervention moderator, given differences between these waves of ADHD coaches. Coaching patterns should continue to be monitored to inform training standards, such as socializing newer coaches to risk management (i.e., needing professional liability insurance, ensuring adequate training prior to workforce entry, appropriate documentation). There may also be a need to formalize professional boundaries between ADHD coaching and psychotherapy, particularly if longer-serving ADHD coaches may be vulnerable to drift in scope of practice. An important next step would be formally identifying the components and mechanisms of ADHD coaching, and how they compare to therapy for ADHD. Future steps may additionally include qualitative interviews with coaches to clarify responses not captured by qualitative analysis.

Ultimately, this work offers guidance for future coaching guidelines or the implementation of state or national statutory regulations for ADHD coaching. As the future of the ADHD coaching workforce will look more similar to the post-pandemic coaching workforce, who may be underrepresented in the field’s leadership positions, it is important to incorporate their voices as regulatory standards or structures are discussed.

### 4.3 Conclusion

The COVID-19 pandemic heightened the need for ADHD psychosocial services and may have tipped the balance toward more accessible vs. more established mental health services. Pandemic-related societal changes may have reduced barriers to entering the ADHD coaching workforce, opening the door for a surge in this non-clinical and non-evidence-based form of support. Differences between pre-pandemic and post-pandemic coaches are indicative of where the ADHD coaching field is headed and should be reviewed in future public health conversations regarding the ADHD coaching profession (e.g., designing training standards, professional development requirements, developing legal regulatory structures, and clinical consultation). The differences detected herein are also important to consider in research contexts evaluating the effectiveness of routine support delivered in ADHD coaching.

## Data Availability

Publicly available datasets were analyzed in this study. This data can be found here: Open Science Framework [https://osf.io/mqgpf].
