# Supplementary material for: ADHD coaching since the COVID-19 pandemic: Is there a new wave of ADHD coaches?
Source: Front Psychiatry. 2026 Sep 3;17:1841020. doi: 10.3389/fpsyt.2026.1841020 (PMC13582364; doi:10.3389/fpsyt.2026.1841020)
Supplement: Supplementary file 1 [file DataSheet1.pdf]

Supplementary Material

1 Supplementary Tables

1.1 Table 1

Comparisons and Hypothesized Directions of Effect for Study Variables

Comparison	Direction of Effect	Broad Hypothesis Category
Lived Experiences		
Self-identify as having ADHD	Pre < Post	Generational[a]
Received formal ADHD diagnosis	Pre < Post	Generational
Received ADHD coaching as client	Pre < Post	Generational
Family member with ADHD	Pre < Post	Generational
Close friends or romantic partners with ADHD	Pre < Post	Generational
Teaching or working professionally with individuals with ADHD	Pre < Post	Generational
Gender		
Woman (vs all other)	Pre > Post	Generational
Man (vs all other)	Pre < Post	Generational
Age (M [SD])	Pre > Post	Generational
White, non-Hispanic	Pre > Post	Generational
Education Level/Degree (Advanced Degree)	Pre > Post	Generational
Healthcare licensure	Pre > Post	Generational
Job Sector Prior to Coaching		
Education	Pre > Post	Generational
Business	Pre > Post	Generational
Mental health	Pre > Post	Generational
Professional Engagement		
Completed an ACO-endorsed ADHD Coach-Led Training Curriculum	Pre > Post	Professional Engagement[b]
Member of a Professional ADHD Coaching Organization	Pre > Post	Professional Engagement
Delivers coaching across state lines	Pre > Post	Professional Engagement
Delivers coaching across international borders	Pre > Post	Professional Engagement
Delivers coaching in-person	Pre > Post	Professional Engagement

Accepts outside funding (e.g., FSA, HAS, Client's employer, health insurance)	Pre > Post	Professional Engagement
Professional Liability Insurance Coverage	Pre > Post	Professional Engagement
Source of Clients		
Advertisement in online directory	Pre > Post	Professional Engagement
Referrals from healthcare providers	Pre > Post	Professional Engagement
Social media marketing	Pre < Post	Generational
Referrals from educational sector	Pre > Post	Professional Engagement
Paid advertisements	Pre > Post	Professional Engagement
Word of mouth	Pre > Post	Professional Engagement
Populations Served		
College students	Pre > Post	Professional Engagement
Adolescents (age 12-17)	Pre > Post	Professional Engagement
Children under 12	Pre > Post	Professional Engagement
I mostly serve White individuals	Pre > Post	Professional Engagement
I mostly serve women/girls	Pre > Post	Professional Engagement
I mostly serve adults with at least a Bachelors	Pre > Post	Professional Engagement
Number of clients on caseload	Pre > Post	Professional Engagement
Length of sessions (minutes)	Pre > Post	Professional Engagement
Number of sessions per month per client	Pre > Post	Professional Engagement
Duration of active services per client (in weeks)	Pre > Post	Professional Engagement
Total weekly direct client hours	Pre > Post	Professional Engagement
Total weekly indirect hours	Pre > Post	Professional Engagement
Individual session rate/fees	Pre > Post	Professional Engagement
Receipt of Supervision or Informal Consultation		
Peer consultation from another coach	Pre > Post	Professional Engagement
Informal consultation from a licensed provider	Pre > Post	Professional Engagement
Clinical supervision from a licensed provider	Pre > Post	Professional Engagement
Conceptualize ADHD as…		
Form of neurodiversity	Pre < Post	Generational
Neurodevelopmental disorder	Pre > Post	Generational
Mental health condition	Pre > Post	Generational
Psychiatric disorder	Pre > Post	Generational
Mental disorder	Pre > Post	Generational

Structured Intervention Materials Utilized	Pre > Post	Session Content
Clinical Concerns: Topics Covered		
ADHD medication adherence	Pre > Post	Session Content
Substance use or addictions	Pre > Post	Session Content
Trauma	Pre > Post	Session Content
Suicide, abuse, and/or harm to self or others	Pre > Post	Session Content
Use of Measurement-Based Care		
Progress monitoring	Pre > Post	Session Content
Interpret outside evaluations	Pre > Post	Session Content
No assessment	Pre < Post	Session Content
Collect client satisfaction ratings	Pre > Post	Session Content
Conducts ADHD diagnostic assessments	Pre > Post	Session Content
Outgoing Referrals to Healthcare		
A (mental) healthcare provider to obtain formal diagnosis	Pre > Post	Session Content
Medication prescribers	Pre > Post	Session Content
Cognitive behavioral therapy providers	Pre > Post	Session Content
Holistic, naturopathic, or other healing arts professionals	Pre > Post	Session Content

[a] Generational Hypothesis: there may be generational differences between pre- and post-pandemic coaches given the migration of ADHD coaching online, and co-occurring links between ADHD-focused lived experience content on social media and advertisements for ADHD coaching (28-30). [b] Professional Engagement Hypothesis: due to the rapid entry of post-pandemic ADHD coaches into the coaching workforce in the context of informal connectedness to the ADHD community through social media, post-pandemic coaches may have lower levels of professional connectedness with other professionals (including healthcare and educational sectors, as well as other ADHD coaches; (31,32). They may additionally report a smaller scale of practice. [c] Session Content Hypothesis: post-pandemic coaches my practice a broader level of content in sessions (including higher coverage of various clinical topics), due to lower socialization to the risks of the coaching field, or due to greater rates of exploring their personal coaching style (33,18).

2 Supplementary Materials

2.1 Data Integrity

In our procedure design, we did not provide compensation and implemented a two-step authentication process to access the survey. Embedded in our survey design, we enabled several features in Qualtrics, including ReCAPTCHA fraud detection, RelevantID duplicate detection, Security Scan Monitor, and Prevent Indexing (34). During the manual data review process, we confirmed that no one completed the survey in less than five minutes and verified and removed duplicate responses

2.2 Participants

Participants were excluded if they had not coached a client residing in the U.S. within the last 12 months (n = 43), and if they self-reported that they had already taken the survey (n = 25). Two participants declined to participate, and 86 participants did not start the survey (this may have included individuals who later returned to take the survey). Removing fraudulent (n = 2) and duplicate (n = 31) responses, as well as participants who did not indicate coaching start date (n = 16), resulted in 465 participants in the present analyses.

2.3 Sensitivity Analysis

Given the statistically significant association between age and coaching start date (Table 1), there was concern that age might act as a confounding variable in the relationship between coaching start date and the study variables. To test this possibility, post-hoc sensitivity analyses were conducted on variables that were initially found to be statistically significant with age added as a covariate. Following sensitivity analysis, most comparisons reported above remained statistically significant, with the exception of the difference in rates ADHD coaches had received a formal ADHD diagnosis, received coaching as a client, or had a family member with ADHD, and of participant race (White, non-Hispanic vs not), which were no longer statistically significant when age was added to the model.
